# Challenges in Translating GWAS Results to Clinical Care

**DOI:** 10.3390/ijms17081267

**Published:** 2016-08-04

**Authors:** Laura B. Scheinfeldt, Tara J. Schmidlen, Norman P. Gerry, Michael F. Christman

**Affiliations:** 1 Institute for Genomics and Evolutionary Medicine, Temple University, Philadelphia, PA 19122, USA; 2 Department of Biology, Temple University, Philadelphia, PA 19122, USA; 3 The Coriell Institute for Medical Research, Camden, NJ 08103, USA; tschmidlen@coriell.org (T.J.S.); norman.gerry@abiolab.com (N.P.G.); christman@coriell.org (M.F.C.); 4 Advanced BioMedical Laboratories, Cinnaminson, NJ 08007, USA

**Keywords:** precision medicine, complex disease, genetic risk

## Abstract

Clinical genetic testing for Mendelian disorders is standard of care in many cases; however, it is less clear to what extent and in which situations clinical genetic testing may improve preventive efforts, diagnosis and/or prognosis of complex disease. One challenge is that much of the reported research relies on tag single nucleotide polymorphisms (SNPs) to act as proxies for assumed underlying functional variants that are not yet known. Here we use coronary artery disease and melanoma as case studies to evaluate how well reported genetic risk variants tag surrounding variants across population samples in the 1000 Genomes Project Phase 3 data. We performed a simulation study where we randomly assigned a “functional” variant and evaluated how often this simulated functional variant was correctly tagged in diverse population samples. Our results indicate a relatively large error rate when generalizing increased genetic risk of complex disease across diverse population samples, even when generalizing within geographic regions. Our results further highlight the importance of including diverse populations in genome-wide association studies. Future work focused on identifying functional variants will eliminate the need for tag SNPs; however, until functional variants are known, caution should be used in the interpretation of genetic risk for complex disease using tag SNPs.

## 1. Introduction

Several factors contribute to health-related quality of life, including: healthcare quality and access, individual behavior and lifestyle choices, environment, and genetics. Many clinical genetic tests have been developed and routinely used for Mendelian disorders, which are generally rare, single-gene disorders [1]; however, the development and deployment of clinical genetic testing for common complex diseases faces many additional challenges due to multifaceted genetic, environmental and behavioral risk factors, and to the limited understanding of functional variation that is assumed to underlie the genetic associations identified in genome-wide association studies (GWAS) [2,3].

Since the sequencing of the human genome and the subsequent genotyping of worldwide population samples, GWAS have become feasible and accessible, and to date over 1000 GWAS have been reported [4,5]. The general goals of GWAS are to identify candidate genes and regions that are associated with disease and disease-related phenotypes to better understand the underlying biology of disease, and to identify genetic risk factors that are associated with disease. Moreover, the majority of GWAS that have been conducted to date have almost exclusively focused on a narrow set of human population samples [3,6,7,8] with two resulting limitations: these studies are likely missing important genetic factors involved in disease, and the extent to which results from these studies will generalize to diverse clinical communities, such as those common in the United States, is an open question.

Here we have designed a study to explore the later limitation of generalizability of genetic risk factors for two common complex diseases in which genetic risk has been previously shown to motivate health behaviors [9,10]. The Coriell Personalized Medicine Collaborative (CPMC) is a prospective research study that began in 2007 and is focused on evaluating the potential clinical utility of personalized risk reports for complex disease and drug response [11,12,13]. Two complex disease risk reports, coronary artery disease (CAD) and melanoma, have been evaluated for the potential to motivate health behaviors [9,10]. Both personalized risk reports consist of non-genetic risk factors, and genetic risk estimated from single genetic risk variants, rs1333049 [14] and rs910873 [15], respectively. Personalized risk reports for both of these diseases (Figures S1 and S2 display example reports) have been delivered to and viewed by thousands of CPMC research participants, and many of these participants have also completed outcome surveys that include information on self-reported behavior changes after report viewing as well as self-reported motivations for behavior change. In both cases, our previous work has shown a significant increase in healthy behavior change after viewing risk reports in the subset of research participants that also reported having increased genetic risk for the disease [9,10].

Given that there is self-reported behavioral data suggesting that genetic risk for CAD and melanoma has the potential to motivate healthy behavior change, it is critical to insure that reported genetic risk is accurately capturing disease risk. In order to evaluate the accuracy of the genetic variants used in risk assessment for CAD and melanoma across diverse human populations, we have leveraged the publicly available Phase 3 1000 Genomes Project whole genome sequencing data [16] and performed a simulation study to test how often reported genetic risk factors are likely to correctly measure actual genetic risk variants.

## 2. Results

We have focused on the performance of the two single nucleotide polymorphisms (SNPs) that have been previously shown to be associated with CAD (rs1333049 [14]) and melanoma (rs910873 [15]), have been used in CPMC personalized risk reports [9,10], and have been shown to motivate heath behaviors [9,10]. Both SNPs have relatively strong effect sizes relative to what is commonly identified in GWAS. More specifically, rs1333049 heterozygotes and rs1333049 homozygotes have relative risks of 1.3 and 1.7, respectively; and rs910873 heterozygotes and rs910873 homozygotes have relative risks of 1.7 and 3.0, respectively. rs1333049 is not present in a gene, and as previously described, the association signal spans over 80 kb [14]. There are several genes in the region surrounding rs910873, and as noted previously, the association signal spans a 400 kb region that contains multiple genes, including *PIGU*, which is the gene containing rs910873 [15]. The risk allele frequencies of rs1333049 (C) and rs910873 (T) range from 0.21 to 0.54 and from 0.00 to 0.05, respectively across the 1000 Genomes population samples [16].

We evaluated the performance of these SNPs as tag or proxy SNPs for what are assumed to be underlying functional variants that are in linkage disequilibrium (LD) with the tag SNP. Using the Phase 3 1000 Genomes Project sequence data surrounding each SNP, we defined four distance windows (5, 10, 50, and 100 kb) and simulated a “functional” variant located within each window. In each population sample (Shown in Figure S3 and described in Table S1), we asked whether the tag SNP was accurately tagging (*R*^2^ ≥ 0.8) the simulated functional variant in each of the 26 1000 Genomes population samples. The simulated “functional” variant was chosen in such a way that the tag SNP had to accurately tag (*R*^2^ ≥ 0.8) it in the CEU (Utah Residents (CEPH) with Northern and Western European Ancestry), which is the 1000 Genomes population sample that most closely resembles the population samples used in the studies that identified the tag SNPs for each disease [14,15], and most closely resembles the majority of population samples that have been included in published GWAS [3,6,7,8]. For each genetic risk variant, we performed 10^6^ independent simulations for each distance window. In total, we performed 8 × 10^6^ simulations.

**Figure 1:**
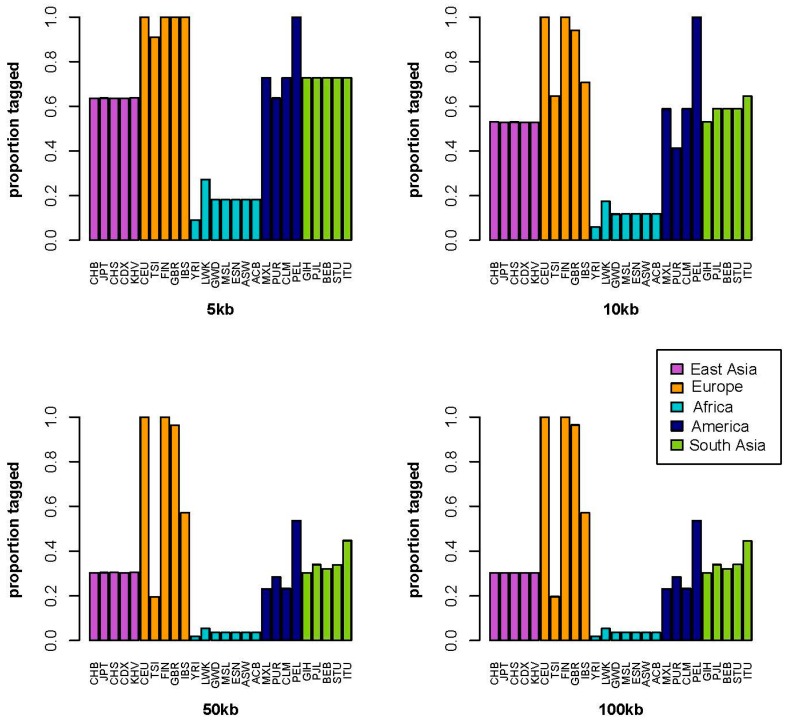
Coronary artery disease (CAD) genetic risk tag single nucleotide polymorphism (SNP) performance. Figure 1 displays four distance windows, each showing the proportion of correctly (*R*^2^ ≥ 0.08) tagged functional variants on the *y*-axis and each 1000 Genomes population sample on the *x*-axis. Population samples are color coded by continental region such that East Asia is purple, Europe is orange, Africa is turquoise, America is dark blue, and South Asia is green. Explanation of population sample abbreviations for the *x*-axis are shown in Table S1.

Figure 1 displays the results for the CAD risk variant for each of the four distance windows. For the smallest distance window, 5 kb, there is reasonably good consistency in tagging among European population samples. The Toscani in Italia population sample is the only European population sample in which the reported tag SNP does not accurately tag the simulated functional variant in every simulation, but rather in 91% of the simulations. The tag SNP performed with lower accuracy in all of the other regional population samples, tagging the “functional” variant in 73% of the simulations across South Asian population samples; 64%–100% of the simulations across Native American population samples; 64% of the simulations across East Asian population samples; and 10%–27% across African and African American population samples.

As the distance window increases, the simulated accuracies decrease. The tag SNP reliably tagged the “functional” variant in 65%–100% of the simulations across European population samples; 53%–65% of the simulations across South Asian population samples; 41%–100% of the simulation across Native American population samples; 53% of the simulations across East Asian population samples; and 6%–18% across African and African American population samples. By the 50 kb distance window, the tag SNP is tagging the simulated functional variant less than 50% of the time, in every population sample except for the Peruvians from Lima, Peru, Iberian Population in Spain, Finnish in Finland, and British in England and Scotland. For the 5, 10, 50, and 100 kb distance windows, the number of potential “functional” SNPs are 11, 17, 56, and 56, respectively.

**Figure 2:**
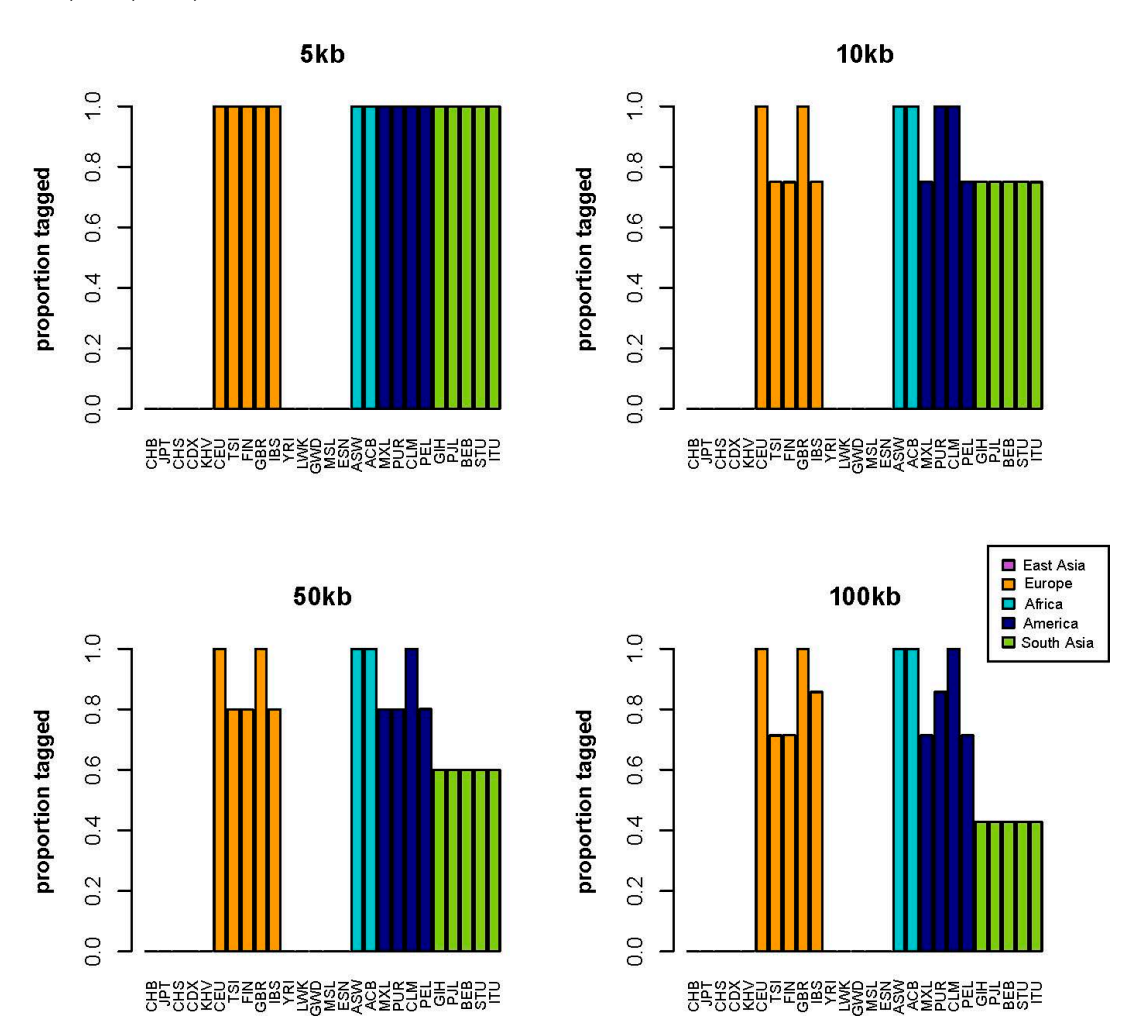
Melanoma genetic risk tag SNP performance. Figure 2 displays four distance windows, each showing the proportion of correctly (*R*^2^ ≥ 0.08) tagged functional variants on the *y*-axis and each 1000 Genomes population sample on the *x*-axis. Population samples are color coded by continental region such that East Asia is purple, Europe is orange, Africa is turquoise, America is dark blue, and South Asia is green. Explanation of population sample abbreviations for the *x*-axis are shown in Table S1.

Figure 2 displays the results for the melanoma risk variant for each of the four distance windows. One aspect of this case study is that the tag SNP is not polymorphic in any of the East Asian or African population samples, which means that there is no information contained in this tag SNP regardless of the distance window for these peoples. For all of the other population samples, at 5 kb, the tag SNP is tagging all of the simulated “functional” variants in the distance window. At 10 kb, tagging is reduced to less than 80% in three of the four European population samples, two of the four Native American population samples, and all of the South Asian population samples. We note that for the 50 kb distance window, tagging actually increases in several population samples. We believe the pattern across distance windows for the melanoma risk variant is due to the relatively small number of possible “functional” SNPs present in the region. For 5, 10, 50, and 100 kb, the number of potential “functional” SNPs are 1, 4, 5, and 7, respectively. Therefore, for the 5 kb window, there is one possible “functional” SNP that is either tagged 100% of the time or 0% of the time across population samples. For the 10 kb window, there are 4 possible “functional” SNPs that can be tagged, so the possible tagging percentages are: 0%, 25%, 50%, 75% or 100%.

## 3. Discussion

For two common complex diseases, CAD and melanoma, we have found that a significant proportion of participants with increased genetic risk self-report increased healthy behavior change (heart health and sun protective behaviors, respectively) [9,10]. These participants also tend to self-report that genetic risk was a motivating factor in their reported behavior change [9,10]. Given that the CPMC does not evaluate clinical end points, we interpret these results to support personalized genetic risk as a potential motivational tool for healthy behavior change. This interpretation is also supported by a study of smoking cessation [17] and by a minority subset (obesity, breast cancer, and rheumatoid arthritis) of evaluated complex diseases in a recent meta-analysis [18]. However, we also note that previous work has not found significant results when generalizing across complex diseases [18]. We suggest that these studies may have been underpowered to identify relationships between behavior change and personalized genetic risk given that they included research participants with no increased genetic risk in their experimental sample. We also suggest that personalized genetic risk may only motivate behavior change to mitigate risk for some but not all complex diseases. If the preliminary self-reported behavioral change motivation results from the CPMC hold over longer time frames (>3 months), then personalized genetic risk factors for at least some complex diseases may be worth utilizing for health behavior motivation. However, this potential is limited by the degree to which genetic risk factors for complex disease are understood and accurate.

Ascertainment bias in published GWAS is a known problem that is expected to limit the relevance of results to persons of non-European descent [3,6]. Here, we have explored the extent to which replicated genetic risk factors for CAD and melanoma generalize across diverse populations. We leveraged the publically available 1000 Genomes whole genome sequencing dataset for 26 worldwide population samples to infer the performance of genetic risk factors for CAD and melanoma as tag SNPs or proxy measurements of simulated “functional” risk variants (i.e., biomarkers of genetic risk for disease). Our results demonstrate that the simulated accuracy of reported genetic risk variants for CAD and melanoma as tag SNPs for underlying functional variation is not encouraging. These results are true across global population samples as well as for population samples of European descent. The extent to which other disease-associated SNPs are located in regions of the genome in which LD varies across population samples will determine the generalizability of our results to other single SNP models of disease risk. However, ascertainment bias may have an even larger negative impact on polygenic models of disease risk where single SNP inaccuracies will likely compound the inaccuracy of the overall model.

Our simulation results therefore suggest that ascertainment bias in GWAS is a serious concern that needs to be addressed. Improving the representation of diverse clinical population samples in GWAS provides many benefits that have already been recognized [8]. Overall, improved representation is likely to contribute to a more comprehensive understanding of all of the genetic risk factors, gene/gene interactions, and gene/environmental interactions that influence complex disease given that important genetic risk factors are likely to be missing from many published studies. This general goal will also facilitate downstream applications such as targeted drug therapies and improved personalized disease risk assessment. Thus, reducing ascertainment bias and improving the quality of results generated from GWAS is likely to improve the accuracy of clinical genetic risk assessment thereby facilitating trust with patients and the general public at large. That is, for complex disease genetic risk information to contribute to health behavior motivation, genetic risk estimates must be accurate and clinically meaningful, and patients will have to trust that the genetic risk estimates are meaningful.

There are several limitations to the current study that should be considered alongside the results presented here. We only included singe SNP genetic risk factors for CAD and melanoma despite the more recent identification of multiple genetic risk factors, and we did not consider the potential impact of SNP imputation on tag SNP performance. However, we anticipate that the incorporation of multiple genetic risk factors and imputation is likely to compound tag SNP performance error rates. In addition, we only considered two complex disease case studies in which existing data demonstrates health behavior motivation potential [19], and results are likely to vary across other diseases.

As many have already noted [2,6,20], enrolling diverse communities in GWAS is an non-trivial challenge, and inclusion of clinical population samples that are more representative of the United States requires the inclusion of marginalized communities that commonly lack access to clinical resources. We applaud the efforts of various funding agencies (including NIH and the UK Wellcome Trust) that currently support GWAS that include diverse peoples and address health disparities and hope that these efforts expand in the future. This support must be structured to permit community outreach and ongoing relationships with marginalized communities (e.g., [21]). We argue that research funding should also support studies that follow-up on the strongest GWAS results with resequencing and functional validation so that the underlying functional variants can be identified and tested directly and replace tag SNPs. Once these functional variants are known, the problematic and error-prone reliance of clinicians on “Race” [22] as a proxy for ancestry in diagnosis and treatment decision making can be improved with individualized health care based on direct measurement of genetic and non-genetic risk factors.

## 4. Materials and Methods

### 4.1. Coriell Personalized Medicine Collaborative

The CPMC is a prospective research study that evaluates the potential clinical utility of genetic risk factors for complex disease. More details on the study can be found in [11,12,13,23,24,25,26]; however, here we will briefly summarize the framework of the study. CPMC scientists and staff use published GWAS to identify replicated SNPs that are associated with complex diseases in at least two independent clinical population samples. These diseases and associated SNPs are evaluated by an external board of experts (ICOB) to determine whether the diseases are potentially actionable, which is defined by the potential for behavior change and/or clinical screening to either mitigate disease risk or contribute to early detection. The disease/SNP sets are also evaluated to insure robust statistical association. The subset of approved disease/SNP sets are then incorporated into personalized risk reports that include genetic and non-genetic risk factors for disease and are periodically provided to research participants through an online web portal. Participants may choose whether or not to view a given personalized risk report, and may optionally complete outcome surveys detailing what if anything they did with the information they chose to view.

### 4.2. Genetic Risk Variants

For the current study we have focused on two complex disease case studies: CAD and melanoma. Personalized risk reports for both of these diseases (see Figures S1 and S2 for example risk reports) were viewed by over 1000 CPMC participants, and both reports were included in the earliest outcome surveys. Previous analysis of these outcome surveys identified significant increases in health behavior change after viewing personalized risk reports in participants that also reported increased genetic risk for the disease [9,10].

### 4.3. Sequencing Data

The data included in the current analysis come from the Phase 3 dataset provided by the 1000 Genomes Project [16]. For all analyses included in the current study, identified related individuals were removed. In addition, one of each pair of unknown relatives present in the 20130606_sample_info_sample_info.csv file downloaded from the 1000 Genomes website were also removed. The 20130502 version of the Phase 3 1000 Genomes data was downloaded, and 5 megabases (Mb) surrounding the CAD and melanoma genetic risk variants (rs1333049 and rs910873, respectively) was extracted with VCFtools [27]. Descriptions of the Phase 3 1000 Genomes population samples are included in Table S1, and a corresponding world map is displayed in Figure S3.

### 4.4. Simulation Study

We used the software package PLINK [28] to calculate pairwise *R*^2^ among all of the 1000 Genomes SNPs present within 5 Mb of each tag SNP genetic risk variant for each of the 26 1000 Genomes population samples. For each genetic risk variant, we performed 1 million simulations each for 4 distance bins: 5, 10, 50, and 100 kb. For each simulation and for each distance bin, we randomly chose a simulated “functional” variant within the distance bin that was in LD (*R*^2^ ≥ 0.80) with the reported genetic risk variant in the CEU population sample. We then recorded how often this simulated “functional” variant was also in LD (*R*^2^ ≥ 0.80) with the reported genetic risk variant in the other 25 1000 Genomes population samples.

## 5. Conclusions

In summary, large-scale genomic association studies have the potential to identify genes and genetic regions that harbor risk factors for disease; however, future research efforts should address ascertainment bias in clinical research participants with broader inclusion and follow up association signals to identify the underlying functional variants to mitigate the high error rates estimated for tag SNP performance.

## Supplementary Materials

Supplementary materials can be found at http://www.mdpi.com/1422-0067/17/8/1267/s1.
