# Supplementary material for: Challenges in Translating GWAS Results to Clinical Care
Source: Int J Mol Sci. 2016 Aug 4;17(8):1267. doi: 10.3390/ijms17081267 (PMC5000665; doi:10.3390/ijms17081267)
Supplement: Supplementary file 1 [file ijms-17-01267-s001.pdf]

# Supplementary Materials: Challenges in Translating GWAS Results to Clinical Care

**Laura B. Scheinfeldt, Tara J. Schmidlen, Norman P. Gerry and Michael F. Christman**

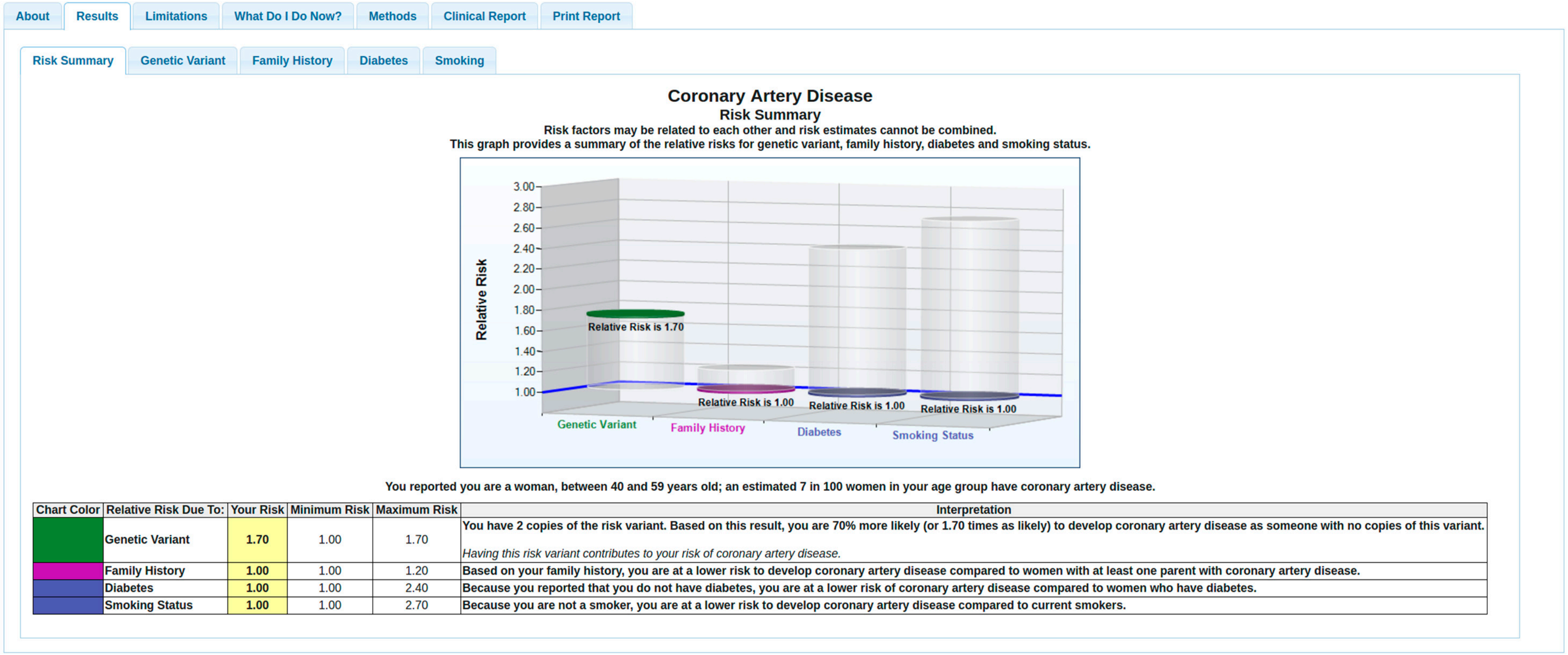


| Chart Color | Relative Risk Due To: | Your Risk | Minimum Risk | Maximum Risk | Interpretation |
|---|---|---|---|---|---|
| | Genetic Variant | 1.70 | 1.00 | 1.70 | You have 2 copies of the risk variant. Based on this result, you are 70% more likely (or 1.70 times as likely) to develop coronary artery disease as someone with no copies of this variant. *Having this risk variant contributes to your risk of coronary artery disease.* |
| | Family History | 1.00 | 1.00 | 1.20 | Based on your family history, you are at a lower risk to develop coronary artery disease compared to women with at least one parent with coronary artery disease. |
| | Diabetes | 1.00 | 1.00 | 2.40 | Because you reported that you do not have diabetes, you are at a lower risk of coronary artery disease compared to women who have diabetes. |
| | Smoking Status | 1.00 | 1.00 | 2.70 | Because you are not a smoker, you are at a lower risk to develop coronary artery disease compared to current smokers. |

**Figure S1.** Example Personalized coronary artery disease (CAD) Risk Report. Figure S1 displays an example Coriell Personalized Medicine Collaborative (CPMC) risk report that displays genetic and non-genetic risk factors for CAD. The Risk Summary includes visualization of risk with the relative risk plotted on the *y*-axis and each risk factor displayed on the *x*-axis. The CAD report includes risk estimated from genetic variant, self-reported family history, self-reported diabetes status, and self-reported smoking status. Numerical relative risks, overall risk ranges, and risk interpretations are included in tabular form.

About | Results | Limitations | What Do I Do Now? | Methods | Clinical Report | Print Report

Risk Summary | Genetic Variant | Family History

## Melanoma

**Risk factors may be related to each other and risk estimates cannot be combined.**
**This graph provides a summary of the relative risks for one genetic variant and family history.**

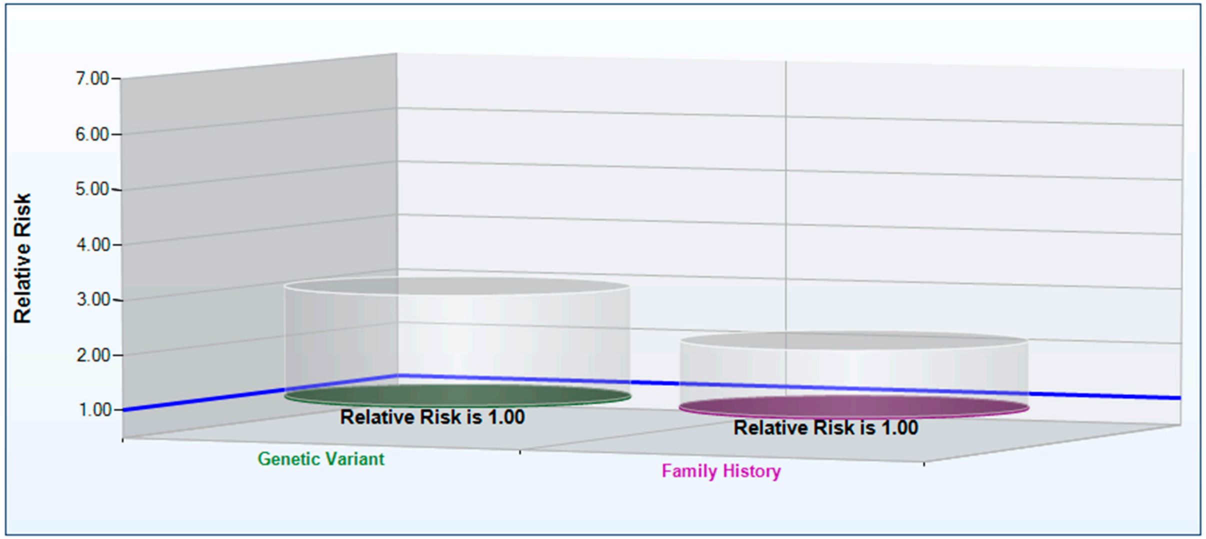


**You reported you are a woman of unknown race/ethnicity, between 40 and 49 years old; data for women of mixed or unknown race/ethnicity in your age group is not available, however, an estimated 3 in 1,000 Caucasian women in your age group have melanoma.**

| Chart Color | Relative Risk Due To: | Your Risk | Minimum Risk | Maximum Risk | Interpretation |
|---|---|---|---|---|---|
| | Genetic Variant | 1.00 | 1.00 | 3.00 | You have 2 copies of the non-risk variant. Based this result, you are at lower risk to develop melanoma compared to someone with one or two copies of this variant. |
| | Family History | 1.00 | 1.00 | 2.20 | Based on your family history, you are at a lower risk to develop melanoma compared to someone with a first degree relative (parent, sibling, or child) with melanoma. |

**Figure S2.** Example Personalized Melanoma Risk Report. Figure S2 displays an example CPMC risk report that displays risk factors for Melanoma. The Risk Summary includes visualization of risk with the relative risk plotted on the *y*-axis and each risk factor displayed on the *x*-axis. The Melanoma report includes risk estimated from genetic variant and self-reported family history. Numerical relative risks, overall risk ranges, and risk interpretations are included in tabular form.

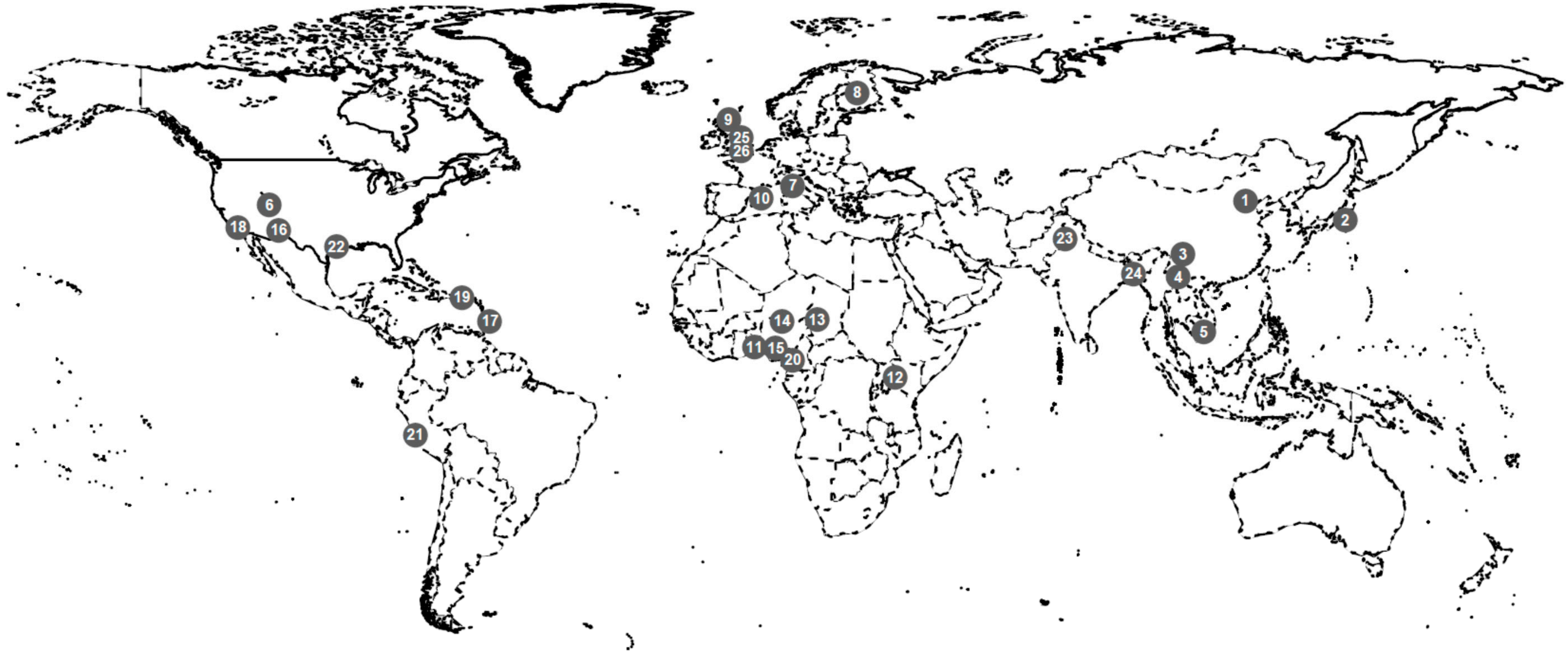


**Figure S3.** World Map of Phase 3 1000 Genomes Population Samples. Figure S1 displays each of the 26 population samples included in the Phase 3 1000 Genomes data. The numbers correspond to the descriptions that are listed in Table S1.

**Table S1.** Phase 3 1000 Genomes Population Samples.

| ID | Code | Population Sample Description |
|---|---|---|
| 1 | CHB | Han Chinese in Bejing, China |
| 2 | JPT | Japanese in Tokyo, Japan |
| 3 | CHS | Southern Han Chinese |
| 4 | CDX | Chinese Dai in Xishuangbanna, China |
| 5 | KHV | Kinh in Ho Chi Minh City, Vietnam |
| 6 | CEU | Utah Residents (CEPH) with Northern and Western European Ancestry |
| 7 | TSI | Toscani in Italia |
| 8 | FIN | Finnish in Finland |
| 9 | GBR | British in England and Scotland |
| 10 | IBS | Iberian Population in Spain |
| 11 | YRI | Yoruba in Ibadan, Nigeria |
| 12 | LWK | Luhya in Webuye, Kenya |
| 13 | GWD | Gambian in Western Divisions in the Gambia |
| 14 | MSL | Mende in Sierra Leone |
| 15 | ESN | Esan in Nigeria |
| 16 | ASW | Americans of African Ancestry in SW USA |
| 17 | ACB | African Caribbeans in Barbados |
| 18 | MXL | Mexican Ancestry from Los Angeles USA |
| 19 | PUR | Puerto Ricans from Puerto Rico |
| 20 | CLM | Colombians from Medellin, Colombia |
| 21 | PEL | Peruvians from Lima, Peru |
| 22 | GIH | Gujarati Indian from Houston, Texas |
| 23 | PJL | Punjabi from Lahore, Pakistan |
| 24 | BEB | Bengali from Bangladesh |
| 25 | STU | Sri Lankan Tamil from the UK |
| 26 | ITU | Indian Telugu from the UK |
